# Modulation of expression of genes involved in glycosaminoglycan metabolism and lysosome biogenesis by flavonoids

**DOI:** 10.1038/srep09378

**Published:** 2015-03-23

**Authors:** Marta Moskot, Joanna Jakóbkiewicz-Banecka, Anna Kloska, Elwira Smolińska, Paweł Mozolewski, Marcelina Malinowska, Michał Rychłowski, Bogdan Banecki, Grzegorz Węgrzyn, Magdalena Gabig-Cimińska

**Affiliations:** 1 Laboratory of Molecular Biology (affiliated with the University of Gdańsk), Institute of Biochemistry and Biophysics, Polish Academy of Sciences, Wita Stwosza 59, 80-308 Gdańsk, Poland; 2 Department of Molecular Biology, University of Gdańsk, Wita Stwosza 59, 80-308 Gdańsk, Poland; 3 Department of Molecular Virology, Intercollegiate Faculty of Biotechnology UG-MUG, Kładki 24, 80-822 Gdańsk, Poland; 4 Department of Molecular and Cellular Biology, Intercollegiate Faculty of Biotechnology UG-MUG, Kładki 24, 80-822 Gdańsk, Poland

## Abstract

Flavonoids were found previously to modulate efficiency of synthesis of glycosaminoglycans (GAGs), compounds which are accumulated in cells of patients suffering from mucopolysaccharidoses (MPSs). The aim of this work was to determine effects of different flavonoids (genistein, kaempferol, daidzein) used alone or in combinations, on expression of genes coding for proteins involved in GAG metabolism. Analyses with DNA microarray, followed by real-time qRT-PCR revealed that genistein, kaempferol and combination of these two compounds induced dose- and time-dependent remarkable alterations in transcript profiles of GAG metabolism genes in cultures of wild-type human dermal fibroblasts (HDFa). Interestingly, effects of the mixture of genistein and kaempferol were stronger than those revealed by any of these compounds used alone. Similarly, the most effective reduction in levels of GAG production, in both HDFa and MPS II cells, was observed in the presence of genistein, keampferol and combination of these compounds. Forty five genes were chosen for further verification not only in HDFa, but also in MPS II fibroblasts by using real-time qRT-PCR. Despite effects on GAG metabolism-related genes, we found that genistein, kaempferol and mixture of these compounds significantly stimulated expression of TFEB. Additionally, a decrease in MTOR transcript level was observed at these conditions.

Mucopolysaccharidoses (MPSs) are autosomal, or X-linked (type II) recessive lysosomal storage disorders caused by the deficiency in activity of a lysosomal enzyme involved in catabolism of glycosaminoglycans (GAGs)^1^. Accumulation of GAGs leads to severe clinical symptoms and significantly shortened life span because of damage of affected tissues and organs, including the heart, respiratory system, bones, joints and, in most MPS types and subtypes, also central nervous system (CNS).

Over the past two decades, several approaches for the cure of MPS diseases have been proposed, each with a number of limitations, however^2^,^3^. Therapies used in a relatively large fraction of MPS patients, such as cells' transplantations (CT) and enzyme replacement therapy (ERT), are notably ineffective for neurological symptoms, the letter due to the poor distribution of enzyme in the central nervous system (CNS)^4^. Obstacles to effective therapies for MPS determine the need for continuous studies in order to enhance therapeutic strategies.

One of such strategies is the implementation of the non-enzymatic substrate reduction therapy (SRT) using GAG metabolism modulators, such as various flavonoids. We demonstrated previously that natural flavonoids, such as genistein (5,7-dihydroxy-3-(4-hydroxyphenyl)chromeon-4-on), kaempferol (3,5,7-trihydroxy-2-(4-hydroxyphenyl)chromen-4-one) and daidzein (7-hydroxy-3-(4-hydroxyphenyl) chromen-4-one) significantly inhibited synthesis and reduced levels of GAGs in cultures of fibroblasts and in MPS mice^5^,^6^,^7^,^8^. Those results are in agreement with data published by others, who reported GAG storage correction in cells derived from patients suffering from either mucopolysaccharidoses type IIIA or VII, after treatment with various isoflavones (a subgroup of flavonoids)^9^. Moreover, combinations of various compounds resulted in more effective reduction of cellular GAG accumulation than the use of any of these flavonoids alone^5^,^9^.

As flavonoids can cross the blood-brain barrier, considering them as compounds potentially useful in the optimization of SRT for neuronopathic forms of MPSs appears reasonable. These compounds are known to exhibit biological activity through inhibition of various kinases^10^, however, the mechanism of action of flavonoids as therapeutic agents for MPS treatment remained unclear. Although genistein was believed to inhibit GAG synthesis by blocking the tyrosine kinase activity of the epidermal growth factor receptor (EGFR)^11^, effects of other flavonoids were found to be independent on this mechanism^5^. It was demonstrated that, contrary to genistein, other flavonoids were not effective in inhibiting EGFR phosphorylation^5^, however, the exact mechanism of action of flavonoids as genetic regulators of GAG turnover remains to be elucidated. Recent findings provided information on a putative genistein targetome responsible for impairment of synthesis, and more importantly, lysosomal enhancement of degradation of GAGs by transcription factor EB (TFEB)^12^. This may be important in the light of contradictory conclusions from different studies regarding effects of genistein on GAG synthesis and accumulation in MPS and mucolipidosis type II (ML II) cells, in which either inhibitory^8^,^9^,^13^; or stimulatory^14^ action of this isoflavone was reported. On the other hand, one may assume that elucidation of the mode of other flavonoids' action on GAG metabolism can be helpful in solving these contradictions completely. Moreover, understanding the mechanism of correction of cellular GAG levels by these components and their mixtures may contribute to potential implementation of them as possible drugs for mucopolysaccharidoses, especially those with neurological symptoms.

## Methods

### Cell lines, culture media and reagents

Human Dermal Fibroblasts adult (HDFa) were purchased from the Cascade Biologics (Portland, USA), while MPS II fibroblasts were obtained from the Children's Memorial Health Institute (Warsaw, Poland). Cells were grown in Dulbecco's modified Eagle's medium (DMEM, Sigma-Aldrich, Steinheim, Germany) supplemented with 10% fetal bovine serum (FBS) and 1% antibiotic/antimycotic solution (Sigma-Aldrich, Steinheim, Germany) at 37°C in a humidified atmosphere containing 5% carbon dioxide (CO_2_). Genistein was synthetized at the Pharmaceutical Research Institute (Warsaw, Poland), while kaempferol and daidzein were obtained from Sigma-Aldrich (Steinheim, Germany). All flavonoids were dissolved in dimethyl sulfoxide (DMSO) and added in the indicated final concentrations as determined in previous studies^5^,^8^ to cell cultures. Cells were seeded to a confluence of approximately 80% and grown for 24 h. Then, the medium was substituted with either flavonoid-free one, containing DMSO at a final concentration of 0.05% (control, Ctrl), or the one supplemented with appropriate amounts of tested flavonoids. Glucosamine, D-[1-3H] hydrochloride (3H-GlcN) was purchased from Hartmann Analytic GmbH (Braunschweig, Germany) and Quant-iT™ PicoGreen® dsDNA Reagent was from Life Technologies (Stockholm, Sweden).

### Total RNA extraction

The High Pure RNA Isolation Kit from Roche Applied Science (Indianapolis, USA) was utilized for total RNA extraction from cells according to the manufacturer's instructions. The quality and quantity of each RNA sample was evaluated using the RNA 6000 Nano Assay on the Agilent 2100 Bioanalyser (Agilent Technologies Inc., USA).

### Microarray processing and real-time quantitative RT-PCR assays for mRNA analysis

Illumina's Human HT-12v4 Expression BeadChips (Illumina Inc., USA) were used for the microarray analysis of three biological replicates. The assay performance, data extraction and statistical analysis were performed as previously described^12^. Gene expression data have been deposited in the NCBI's Gene Expression Omnibus (GEO, http: www.ncbi.nlm.nih.gov/geo, GEO Series accession number GSE43692), according to the MIAME standards. Gene Ontology (GO) tool was used in enrichment analysis together with GOrilla applied for identification and visualization of enriched GO terms in ranked lists of genes, and Gene Set Enrichment Analysis (GSEA) for interpreting biological meaning of defining gene sets^15^,^16^.

LightCycler® System 480 (Roche Applied Science, Indianapolis, USA) was used for real-time quantitative Reverse Transcription PCR (real-time qRT-PCR) performance. For cDNA synthesis, 0.5 *µ*g of total RNA was reverse transcribed using the Transcriptor First Strand cDNA Synthesis kit (Roche Applied Science, Indianapolis, USA). Real-time qRT-PCR was performed using Real Time ready Custom Panel (cat no. 05532914001, config. no. 90014731, Roche Applied Science, Indianapolis, USA) and LightCycler® 480 Probes Master (Roche Applied Science, Indianapolis, USA) according to the manufacturer's instructions.

For both, microarray and real-time qRT-PCR analyses, a fold change (FC) greater or equal to 2.0 or 1.3, and below 0.5 or 0.7, for whole genome or GAG associated transcripts, respectively, was considered as a relevant criterion for genes being significantly differentially expressed.

### Statistical analyses of microarray and qRT-PCR data

Each experiment of microarray as well as real-time qRT-PCR analyses was repeated at least three times (n). Data are reported as the mean ± SD with p < 0.05 considered statistically significant. Statistical analysis of *TFEB* expression made via real-time qRT-PCR was performed using ANOVA with Tukey's HSD Post Hoc test with p < 0.005.

### Cell number and viability assessment

HDFa and MPS II fibroblasts' cells were seeded in triplicate in 6-well plates at a density of 10^4^ per well. The medium was changed the following day and supplemented with appropriate amounts of tested flavonoids, or 0.05% DMSO as control, for 24, 48 or 72 hours. Cells were counted and viability was estimated by MUSE® Cell Analyzer (Merck Millipore, Germany) and Muse® Count & Viability Assay Kit (Merck Millipore, Germany). An average of 2000 cells was analyzed for each condition.

### Measurement of kinetics of GAG synthesis

Kinetics of GAG was estimated by measurement of incorporation of glucosamine, D-[1-3H] into GAG chains. In brief, cells were plated in a number of 2 × 10^4^ per well in 48-well plate and incubated overnight to allow the attachment. Next, cells were preincubated for 48 hours in standard DMEM supplemented with appropriate amounts of flavonoids or DMSO (control cultures) and then cells were labelled for 24 hours with 10 *µ*Ci/ml of 3H-GlcN in DMEM without glucose and pyruvate supplemented with 10% FBS and flavonoids or DMSO. After labelling, cells were washed six times with PBS and digested for 3 hours at 65°C with 0.5% papain (prepared in 200 mM phosphate buffer (Na_2_HPO_4_ - NaH_2_PO_4_), pH 6.4, containing 100 mM sodium acetate, 10 mM Na_2_EDTA and 5 mM L-cysteine). Incorporation of 3H-GlcN was measured in MicroBeta2 scintillation counter [PerkinElmer] and Quant-iT™ PicoGreen® dsDNA Reagent was used to determine DNA concentration in papain digested samples. Incorporation of 3H-GlcN was calculated per DNA amount (cpm/ng of DNA) and expressed as relative to control cultures (treated with DMSO alone). To test the efficiency of flavonoids on kinetics of GAG synthesis, one-way ANOVA was performed with Tukey's multiple comparisons test as a post-hoc comparator with significance declared at *p* < 0.05.

### Assessment of GAG levels

Accumulation of GAGs was estimated with Alcian Blue reagent using sGAG quantitative kit WIESLAB®. In brief, confluent cells were plated in a number of 1.5 × 10^5^ per well in 6-well plate and incubated overnight to allow the attachment. Next, cells were supplemented with standard DMEM with appropriate amounts of flavonoids or DMSO (control cultures). The medium was replaced every 5 days. After 10-days of incubation harvested cells were digested with 0.5% papain (see GAG kinetics experiment) and GAG content with Alcian Blue and DNA concentration with PicoGreen were estimated according to manufacturer's protocols. GAG content was per DNA amount (µg/µg of DNA) and normalized to control cultures (treated with DMSO alone).

## Results

### Selection of flavonoids and their cytotoxic activity

It has been demonstrated previously that genistein (an isoflavone), kaempferol (a flavonol) and daidzein (an isoflavone) are among flavonoids which inhibit GAG synthesis considerably in cultured MPS fibroblasts, permitting significant reduction in lysosomal storage^5^,^8^,^9^. Moreover, mixtures of those compounds, i.e. genistein plus kaempferol, and genistein plus daidzein, resulted in even more effective reduction of GAG accumulation than the use of any of these flavonoids alone^5^. Apart from genistein, previously investigated in the context of lysosomal modulation^12^, in this study, the above mentioned two compounds, as well as their mixtures, were selected and applied for further transcriptomic network analysis.

**Figure 1:**
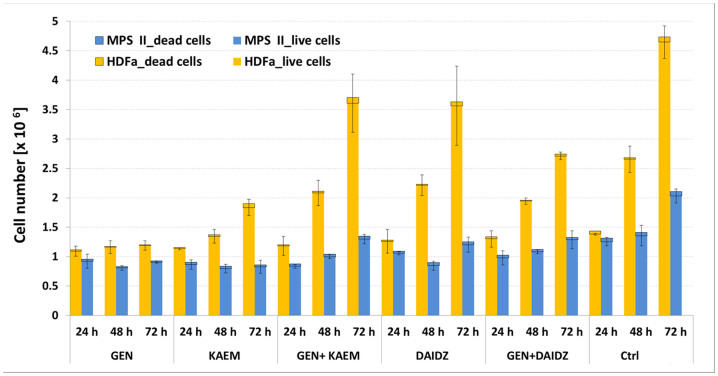
Determination of count and viability of HDFa and MPS II cells respectively with 100 *μ*M of genistein, 100 *μ*M of kaempferol, 100 *μ*M of daidzein, mix of genistein and kaempferol or genistein and daidzein (30 *µ*M each), and with 0.05% DMSO (Ctrl) for different periods of time (24, 48 and 72 h). Data are represented as mean and bars show SD values of experiments run in triplicate.

To examine the cytotoxicity effect of selected flavonoids and mixtures of those compounds we measured viability of HDFa and MPS II fibroblasts treated with 100 *μ*M of genistein, 100 *μ*M of kaempferol, 100 *μ*M of daidzein, mix of genistein and kaempferol or genistein and daidzein (30 *µ*M each), and with 0.05% DMSO (control) for different periods of time (24, 48 and 78 h). As seen in Figure 1 the cell viability did not change remarkably at the tested conditions.

### Effects of flavonoids and their mixtures on GAG synthesis and storage

Because literature contains publications reporting either inhibition of GAG synthesis and resultant decreased storage^5^,^8^,^9^,^13^,^17^ or opposite results^14^ in different cells, we tested effects of flavonoids and mixtures of them in cultures of both HDFa and MPS II fibroblasts. Tested compounds were added individually, i.e. genistein, kaempferol, daidzein at the final concentration of 100 *μ*M, or applied as mix of them, i.e. genistein and kaempferol or genistein and daidzein (30 µM each) to cell cultures. Synthesis of GAGs was measured by estimation of the amount of incorporated precursor, glucosamine, D-[1-3H] hydrochloride. We found that all tested flavonoids, except for daidzein, inhibited GAG synthesis significantly in both wild-type and MPS cells (Table 1). The most pronounced impairment of production of GAG was observed in the presence of genistein, keampferol and mixture of these two, relatively to untreated control cells. Therefore, we confirmed inhibitory effects of genistein and keampferol on GAG synthesis in the cells employed in this study.

Effects of flavonoids on GAG storage was measured after 10 days of treatment as changes in GAG levels are slower than effects on synthesis of these compounds. Moreover, recent studies indicated that GAG levels could be modulated by genistein due to stimulation of lysosomal biogenesis^12^. We found a decrease in GAG levels in cells treated with all tested flavonoids, including daidzein which did not decrease GAG synthesis (Table S1). Thus, we conclude that these compounds decrease storage in employed cells, due to inhibition of GAG synthesis or stimulation of lysosomal biogenesis or both.

### Microarray data association

An overview of microarray experiment performance was gained by clustering samples using correlation metric (Illumina® BeadStudio Data Analysis Software). A comparison of differentially treated samples in the global gene expression pattern was performed for all the biological repeats individually as well as together, based on the correlation distance between all samples computed with the Pearson Correlation Coefficient (PCC). We used hierarchical clustering with average linkage as agglomeration rule (data not shown). Dendrograms based on this metric are useful for identifying outliers, as samples with most similar expression profiles determined by correlation value are clustered together. The hierarchical agglomerative clustering identified two main groups, one including group treated for 24 h, and the other treated for 48 h, all containing three replicates. Each group was composed of cells divided into eight subgroups, which are cells treated with kaempferol at concentrations of 30, 60 or 100 *µ*M, with mix of genistein and kaempferol (30 *µ*M each), with daidzein at 60 or 100 *µ*M, with mix of genistein and daidzein (30 *µ*M each), with 0.05% DMSO, and DMSO-treated cells (non-treated control). Next, the reproducibility between replicate samples was assessed also by calculating PCC. The values ranging between 0.97 and 0.99 for biological replicates show a high degree of reproducibility and strong correspondences between expression profiles. These associations may, in the global view, indicate a prevalence of cells with respect to certain treatment effect on gene expression profiles.

### Effects of flavonoids on global gene expression

**Figure 2:**
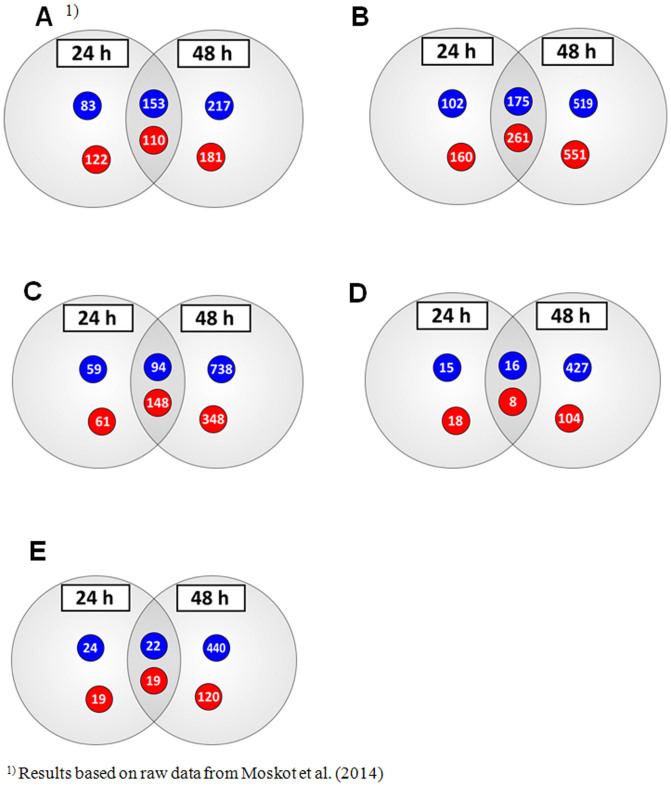
Distribution of up- (red) and down-regulated (blue) transcripts of whole genome of HDFa cells after 24 and 48 h treatment with (A) genistein (100 *µ*M), (B) kaempferol (100 *µ*M), (C) mix of genistein and keampferol (30 *µ*M each), (D) daidzein (100 *µ*M), and (E) mix of genistein and daidzein (30 *µ*M each), at 0.5 ≥ FC ≥ 2 for n ≥ 3, with the p-value < 0.05. DMSO-treated cells were used in control experiments.

**Figure 3:**
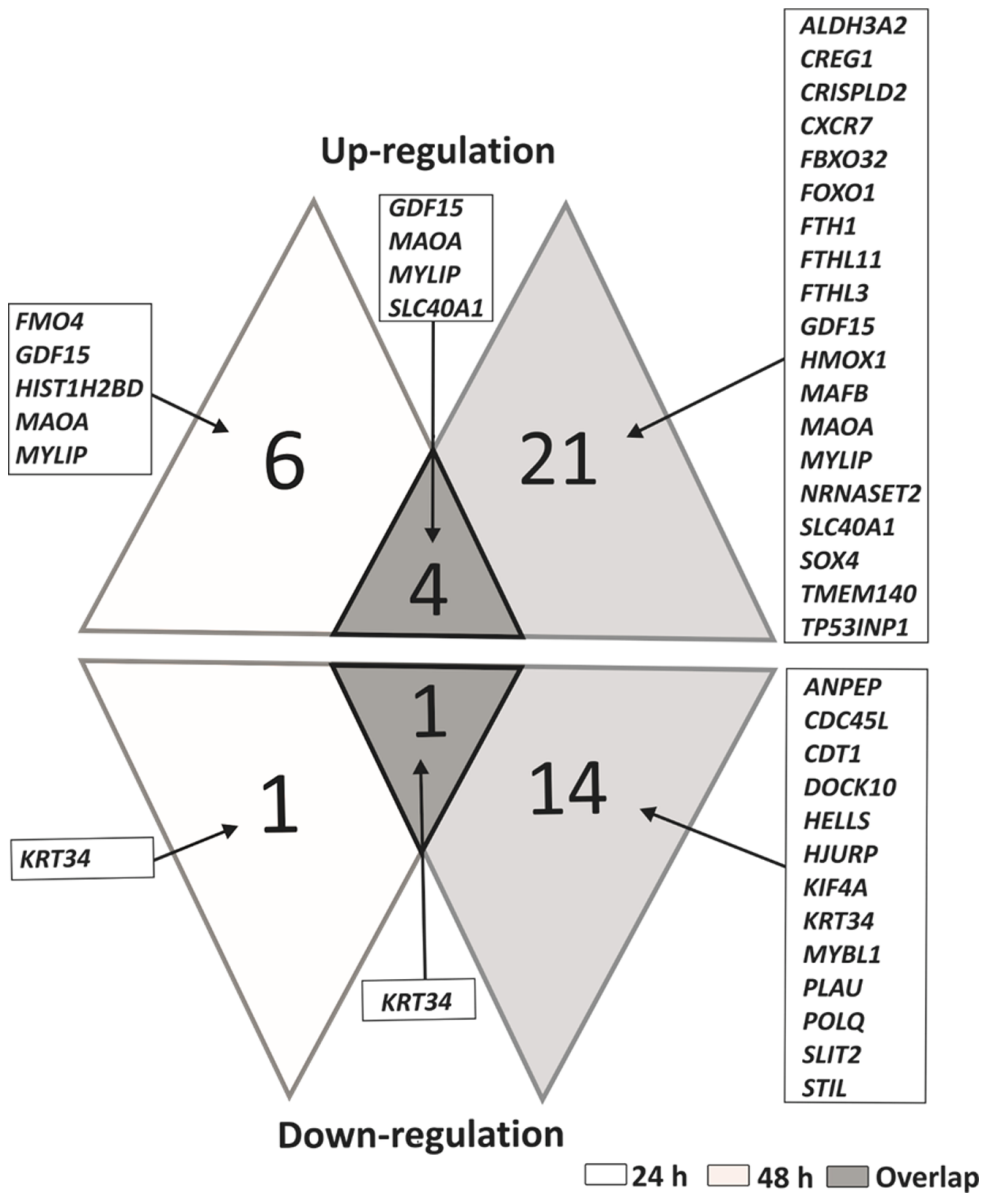
Genes identified as changed under studied conditions with corresponding overlap between the datasets (0.5 ≥ FC ≥ 2, n ≥ 3, with the p-value < 0.05). Up- and down-regulated genes of whole genome of HDFa cells after 24 and 48 h treatment with 100 *µ*M genistein, 100 *µ*M kaempferol, genistein-kaempferol of 30 *µ*M each, 100 *µ*M daidzein and genistein-daidzein of 30 *µ*M each.

Microarray analyses were performed on HDFa cells after 24 and 48 h of either vehicle or 30, 60, or 100 *µ*M compound treatment, or their mixtures, 30 *µ*M each. The detailed studies revealed that the cells responded to different types of treatment with changes in gene expression profiles, affecting large number of genes that showed changes greater than 2-fold (Table 2). Concentration- and time-dependent effects of tested flavonoids on global gene expression in fibroblasts were observed. The highest number of genes with modulated expression was observed for kaempferol and genistein-kaempferol mix treatment type of fibroblasts after both 24 and 48 hours. In total, 698 and 362 for 24 h, and 1506 and 1328 transcripts for 48 h handling with 100 *µ*M kaempferol and mix of genistein with kaempferol (30 *µ*M each), respectively, were affected. Moreover, number of genes exhibiting regulated activity, including both increased and decreased expression, was considerably higher for kaempferol and genistein-kaempferol treated cells than in case of those exposed to genistein alone, as reported by Moskot et al. (2014). In the course of this study, we obtained 436 (100 *μ*M kaempferol), 242 (genistein and kaempferol, 30 *μ*M each), 41 (100 *μ*M daidzein) and 24 (genistein and daidzein, 30 *μ*M each) transcripts, which levels were affected after both 24 and 48 hour treatment time period (Figure 2), while 263 for 100 *μ*M genistein handling of cells as previously described^12^. Among 4 genes that displaced a greater than 2-fold increase in expression after 24 and 48 h at 100 *μ*M kaempferol, genistein-kaempferol of 30 *μ*M each, 100 *μ*M daidzein and genistein-daidzein of 30 *μ*M each, *MAOA* was the only transcript modulated also at 100 *μ*M genistein as reported earlier^12^, while *KRT34* was the only gene down-regulated at all these conditions (Figure 3). *MAOA* and *KRT34* are related to complications associated with various lysosomal storage disease such as mucopolysaccharidoses^12^,^18^,^19^,^20^.

**Figure 4:**
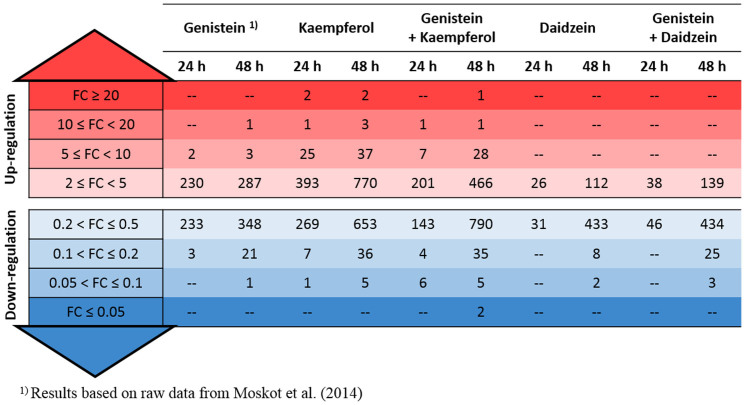
Number of genes whose expression was altered as a function of the FC in response to various flavonoids' treatment type (100 *µ*M genistein, 100 *µ*M kaempferol, 100 *µ*M daidzein, and mixtures of them of 30 *µ*M each), identified in the microarray analysis of whole genome sequences and transcripts of HDFa cells (n ≥ 3, with the p-value < 0.05).

**Figure 5:**
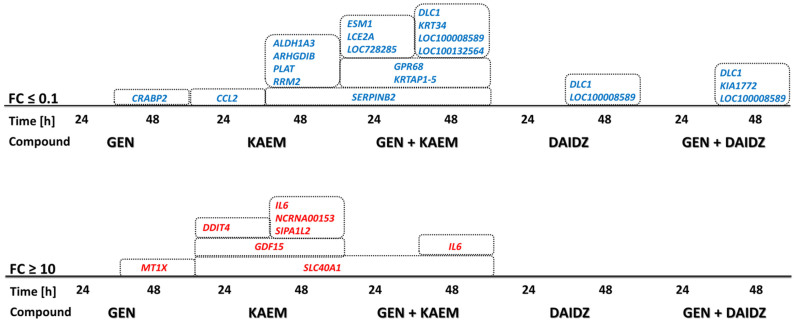
Genes with significantly (i.e. more than 10-fold change) regulated expression upon various flavonoids’ treatment (100 µM genistein - GEN, 100 *µ*M kaempferol - KAEM, 100 *µ*M daidzein - DAIDZ, and mixtures of them of 30 *µ*M each, GEN + KAEM or GEN + DAIDZ) identified in the microarray analysis of whole genome sequences and transcripts of HDFa cells (n ≥ 3, with the p-value < 0.05).

Furthermore, detailed analysis showed a significant, i.e. higher than 10-fold, enhancement of gene expression, mainly in kaempferol and genistein-kaempferol treated cells (Figure 4), for *SLC40A1* mRNA species, up-regulated for both the 24 and 48 hour exposures, and *SLC40A1* and *IL6* transcripts, enriched only after 48 hour time course set (Figure 5).

### Activity of genes involved in GAG metabolism pathways and lysosomal function

It was possible to produce a refined list of genes involved in GAG metabolism that were consistently differentially expressed in HDFa cells treated with various flavonoids and mixtures of them (Table 3). Within this list, there are several genes that were activated by more than one treatment conditions, however, among them only 2 genes, *EXT1* and *HS3ST3A1*, associated with the GAG synthesis pathway, were down-regulated at all tested flavonoids after 24 and 48 hours, respectively. Among genes involved in GAG degradation, *GNS* and *HEXA* were up-regulated after kaempferol and kaempferol-genistein treatment for 48 h. In general, handling of cells with 100 *µ*M kaempferol for 48 h affected the highest number of genes involved in GAG metabolism pathways (i.e. 13 genes of GAG biosynthesis and 7 of GAG degradation) (Table 3).

Our analyses identified also dozens of genes with known roles in lysosomal biogenesis and/or function, which expression was altered by tested flavonoids (partly included in Table 4). These data are in substantial correlation with results reported previously, where it was found that genistein remarkably stimulated genes encoding lysosomal proteins^12^. Furthermore, detailed studies revealed 3 genes (*CLN5* coding for ceroid-lipofuscinosis neuronal protein 5 precursor, *LGMN* coding for legumain and *MANBA* coding for beta mannosidase, all up-regulated after 24 and 48 h) with modulated activity at all tested flavonoids (data not shown).

### Real-time quantitative RT-PCR for mRNA analysis of GAG metabolism- and lysosome- associated genes

We used a real-time quantitative RT-PCR approach to examine in more detail the expression patterns of genes involved in GAG metabolism, as well as in lysosome biogenesis and function, whose activities were modulated in fibroblasts at tested conditions, as observed in microarray studies. Real Time ready Custom Panel covering in total 45 genes, 13 of GAG metabolism pathways (7 of GAG synthesis and 6 of GAG degradation) and 30 sequences coding for lysosomal proteins, all revealing modulated activity in microarray analysis, was designed. The expression of genes was confirmed not only in HDFa cells, but also in MPS II fibroblasts, after 24 h treatment (Table 4), as well as at 48 h period (data not shown), with respect to *TRFC* (transferrin receptor gene), the reference of constant expression level. The same pattern of mRNA level of all verified genes was observed when two other reference genes, *PGK1* and *RPLPO*, were considered (data not shown).

In general, the two independent gene expression profiling methods showed a strong correlation, as for each, similar patterns of expression were observed whether using the microarray or real-time qRT-PCR analysis (Table 4). Similarly to previously published results obtained with genistein-treated cells^12^, here we report that among genes with significantly altered activity in the presence of kaempferol and mixture of genistein-kaempferol, in both HDFa and MPS II fibroblasts treated for 24 h, there are those participating in GAG metabolism pathways (i.e. *EXT1*, *HS3ST3A1* and *XYLT1* involved in anabolism, and *HEXA* in catabolism of GAG). This impact is, however, less pronounced than that reported previously for genistein^12^. Moreover, we confirmed considerable up-regulation of expression of several lysosome-associated genes (i.e. *ACP5*, *AGA*, *AP3S2*, *ARSG*, *CLN3*, *CLN5*, *CTSF*, *LGMN*, *MAN2B1*, *MANBA*, *NEU1*, *NPC1* and *SUMF1*) in response to tested flavonoids after 24 h period of their action (Table 4).

### TFEB and MTOR transcript amount assessment

**Figure 6:**
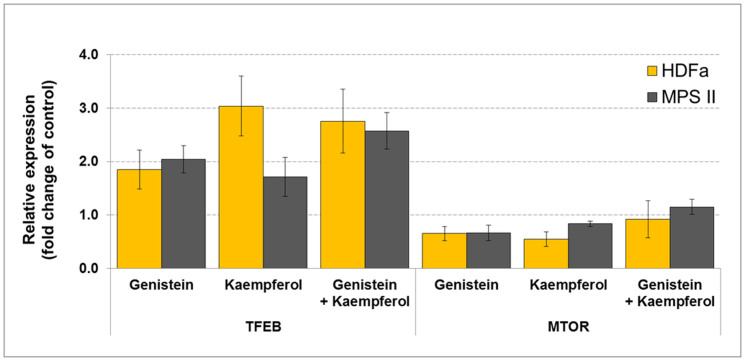
*TFEB* and *MTOR* expression analysis in HDFa and MPS cells via real-time qRT-PCR. Data represent averaged values ± SD from n = 3, and mean significant differences for samples treated for 24 h with various flavonoids (100 *µ*M genistein, 100 *µ*M kaempferol, and mixtures of them of 30 *µ*M each) against non-treated, with respect to endogenous reference gene *GAPDH* (in HDFa) and *RPLPO* (in MPS II), with p < 0.005 as determined by ANOVA with Tukey's HSD Post Hoc.

Interestingly, we found that the tested flavonoids induced an increase in *TFEB* mRNA level and a decrease in *MTOR* (gene coding for Serine/Threonine-Protein Kinase MTOR) transcript amount compared with the untreated samples, in both HDFa and MPS II cells treated for 24 h (Figure 6), in respect to *GAPDH* and *RPLPO*, correspondingly. Qualitatively similar results were obtained for other two reference genes, *TBP* in HDFa, and *TFRC* in MPS II fibroblasts (data not shown).

### Gene Ontology and Gene Set Enrichment analyses

**Figure 7:**
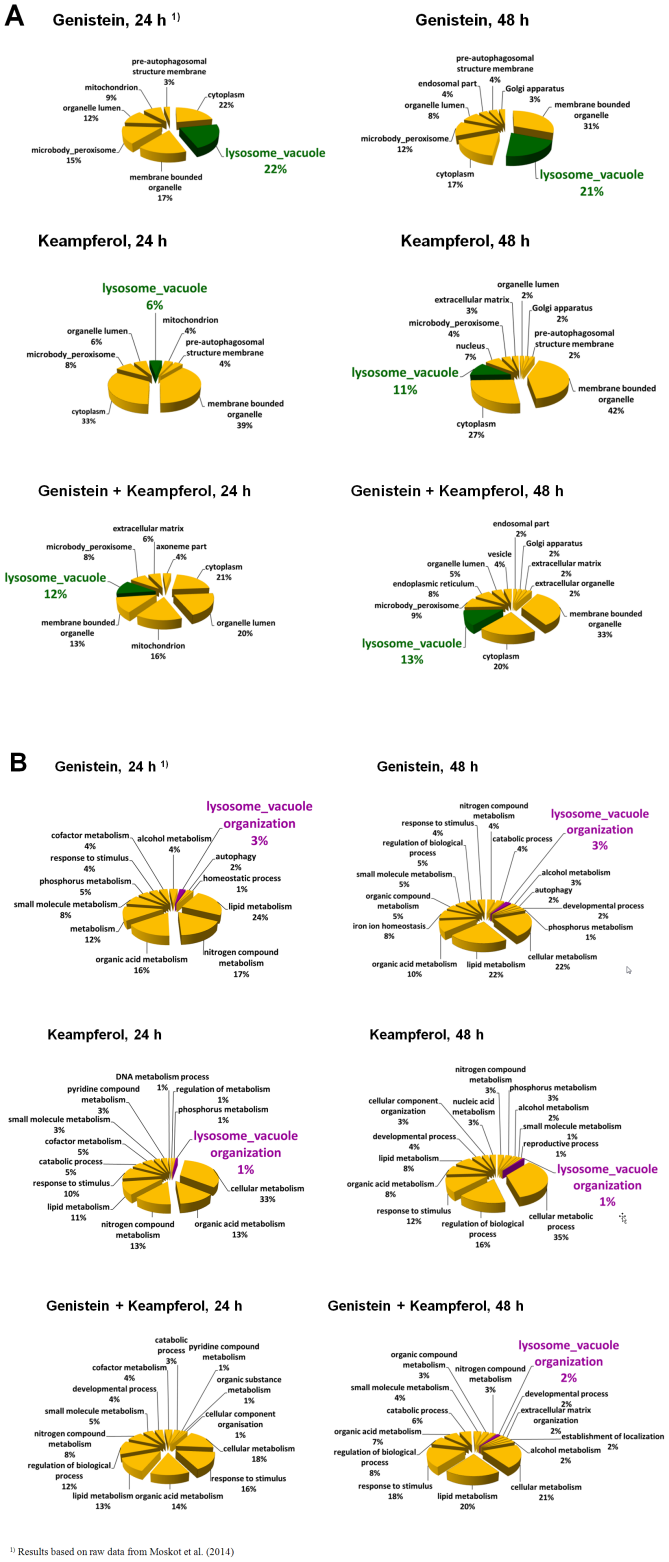
GO analysis by ‘Cellular Compartment’ (A) and ‘Biological Processes’ (B) category of the genes with up-regulated expression upon various flavonoids' treatment (100 *µ*M genistein, 100 *µ*M kaempferol, and mixtures of them of 30 *µ*M each, for 24 and 48 hours) of HDFa cells, with false discovery rate (FDR) < 0.1, fold change ≥ 1.3 and below 0.7, and p < 0.001.

**Figure 8:**
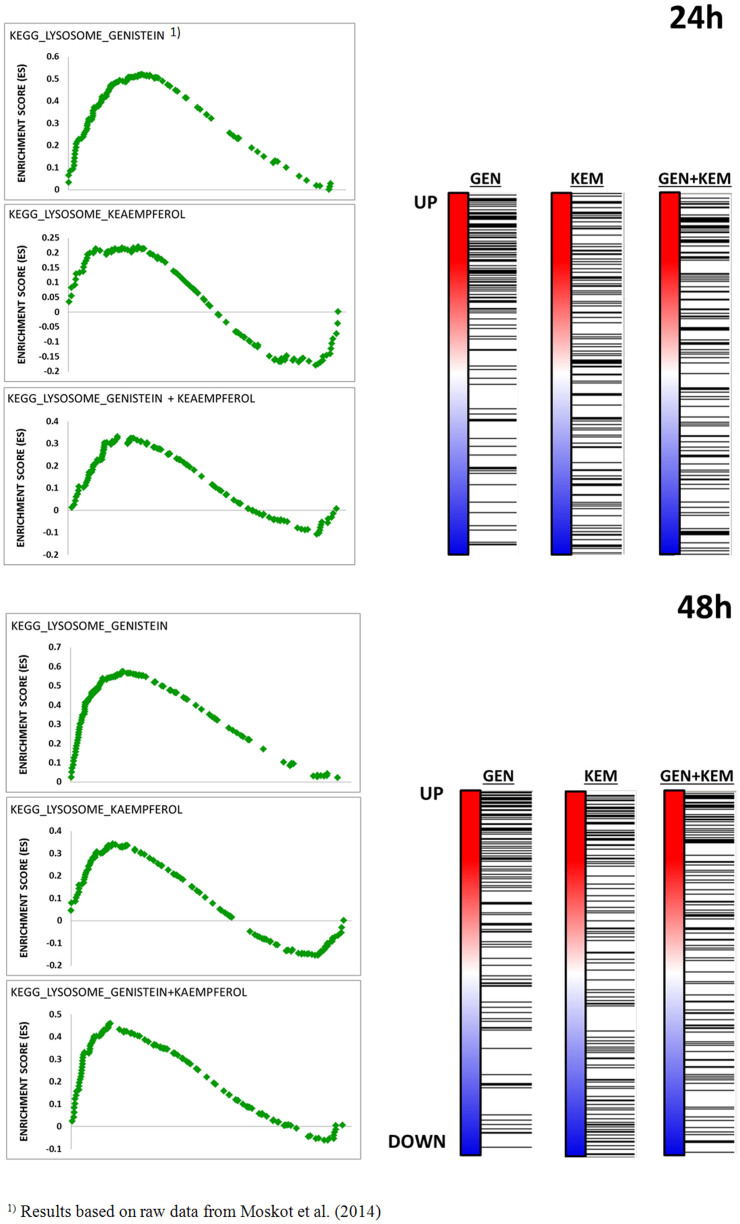
Graphs showing the enrichment plots generated by GSEA analysis of ranked gene expression data (UP: up-regulated, red; DOWN: down-regulated, blue). Sizes of normalized enrichment score (NES), q-value of false discovery rate (FDR q-val), and nominal p-value (NOM p-val) for lysosome-associated gene sets regulated by tested flavonoids (100 *µ*M genistein, 100 *µ*M kaempferol, and mixtures of them of 30 *µ*M each, for 24 and 48 hours) in fibroblasts are depicted. The enrichment score is shown as a scattered green line, and the horizontal black bars next to the plot indicate the position of lysosome-associated genes, which are mostly grouped in the fraction of up-regulated genes.

By selecting informative genes from microarray data via Gene Ontology analysis, we found that all tested conditions altered the expression of genes belonging to a wide range of pathways involved in ‘Cellular Compartment’ organization and ‘Biological processes’ (Figure 7). Network related to lysosome biogenesis and/or function was basically identified for ‘Cellular Compartment’ terms analysis only when samples were treated with mixture of genistein-kaempferol, as well as with kaempferol alone. These enrichments were, however, noticeably weaker than those observed when genistein was applied^12^. Accordingly, Gene Set Enrichment Analysis showed a significant enrichment among these lysosome-associated genes regulated by genistein-kaempferol mix and kaempferol (Table 5), with normalized Enrichment Score (Figure 8), again still weaker than in the case of genistein, as reported previously^12^.

## Discussion

Although all types of mucopolysaccharidoses, inherited metabolic diseases characterized by lysosomal storage of GAGs, are caused by mutations in single genes, their pathomechanisms are more complicated than just accumulation of non-degradable compounds in cells. Although the storage is the primary effect of each MPS-causing mutation, there are various secondary and tertiary effects that lead to a complicated picture of each MPS type and subtype, as well as to a high variability of symptoms among patients suffering from the same disease (for a recent review, see^21^). In the light of this complicated pathomechanism of the disease, it appears crucial to precisely understand mechanisms of actions and effects of any potential drugs that could be used in MPS treatment. One group of such potential drugs are flavonoids, compounds which were reported previously to partially inhibit GAG synthesis and to reduce GAG storage in cells derived from MPS patients. Among them, genistein (an isoflavone) has been studied intensively, and it was proposed that this compound can down regulate GAG production by blocking phosphorylation of the EGF receptor, thus, impairing a signal transduction pathway necessary for activation of genes coding for enzymes involved in this anabolic process^8^,^11^. Nevertheless, other studies indicated that different flavonoids, either natural or synthetic, can also significantly modulate GAG synthesis and storage, while acting through an EGF-independent mode^5^,^9^,^17^. Enhanced effects of combinations of various flavonoids on GAG synthesis and accumulation, relative to single compounds from this group, have also been reported^5^,^9^. Furthermore, recent studies indicated that, at least under certain conditions, genistein might stimulate, rather than inhibit, synthesis of GAGs^14^, which was in contrast to previous reports showing impairment in GAG production and storage in MPS and ML II cells treated with this isoflavone^8^,^9^,^13^. Results of very recent studies led to conclusions that genistein can both inhibit expression of some genes coding for GAG-synthesizing enzymes and stimulate expression of most of genes which products are involved in GAG degradation; the latter effect appears to be due to activation of expression of *TFEB*, a gene coding for transcription factor EB, and transport of this protein into nucleus^12^.

In the light of the above described facts and uncertainness on mechanisms of flavonoid-mediated regulation of GAG metabolism, in this work, the transcriptome of the cell line HDFa was profiled with Illumina's Human HT-12 v4 Expression BeadChips in the presence or absence of various flavonoids or their mixtures. Results of the microarray analyses provided preliminary pictures of influences of tested compounds on cell transcriptome. However, since simultaneous analysis of thousands of genes may potentially lead to either false positive or false negative results, we have repeated analysis of selected genes, which signals in the microarray experiments suggested a significant influence of tested compounds on their expression, by using real-time qRT-PCR. The latter method allows for precise determination of the level of particular transcript, in a quantitative manner. The real-time qRT-PCR experiments involved not only HDFa cells, but also fibroblasts derived from a patient suffering from MPS II.

Transcriptome analyses indicated that, despite certain similarities, there are also significant differences between effects of genistein, kaempferol, and daidzein on global gene expression patterns in human wild-type fibroblasts. The results suggested that these flavonoids may differentially influence GAG metabolism, indeed. This was confirmed by real-time qRT-PCR studies, in which genes coding for proteins involved in GAG synthesis and degradation were investigated in more detail. Importantly, both microarray and real-time qRT-PCR analyses gave similar results, indicating accuracy of both methods. Moreover, changes in expressions of these genes in flavonoid-treated cells, relative to controls, were similar in both wild-type and MPS II fibroblasts. Therefore, we conclude that GAG storage does not influence significantly the response of tested genes to investigated compounds. Interestingly, effects of genistein (an isoflavone) were more similar to those of keampferol (a flavonol) than daidzein (another isoflavone). In fact, daidzein differs from genistein only by a lack of one hydroxyl group, suggesting that this moiety may be crucial in functions of flavonoids as regulators of expression of genes involved in GAG metabolism. This can be assumed on the basis of significantly less pronounced effects of daidzein relative to other flavonoids (Table 1 and 3). On the other hand, despite obvious overlaps, there are also differences in genes which expression is either stimulated or impaired by genistein and kaempferol. Intriguingly, the differences could be observed mostly in expression of genes coding for enzymes involved in GAG synthesis. This might corroborate previous suggestions^5^,^17^ that detailed mechanisms of regulation of these genes by various flavonoids may be different. Most probably different signal transduction pathways are differentially affected by genistein, kaempferol and daidzein, which results in specific transcription patterns of GAG metabolism-related genes in cells treated with these compounds. This hypothesis is supported by earlier findings that genistein, but not kaempferol and daidzein, inhibits phosphorylation of EGF receptor^5^,^11^.

Contrary to genes coding for GAG-synthesizing enzymes, effects of genistein and kaempferol on expression of genes encoding enzymes responsible for GAG degradation were similar. In both cases, transcription of most such genes was stimulated. Daidzein was inactive in this mechanism, as no increase in the level of any GAG degradation-related gene transcript could be detected. Again, a difference in a single chemical moiety results in dramatically different effects of particular flavonoid on expression of genes controlling GAG degradation (Table 3). On the other hand, it is likely that stimulation of expression of these genes by genistein and kaempferol is due to modulation of the same process. Both these flavonoids enhance expression of the gene coding for TFEB (Figure 6), a master regulator for lysosomal biogenesis and function^22^,^23^,^24^, and impair expression of the gene encoding MTOR (Figure 6), a serine/threonine-protein kinase that phosphorylates TFEB, preventing its entering into the nucleus^25^,^26^,^27^,^28^. Interestingly, a combination of genistein and kaempferol did not cause an increase in their effects on TFEB and MTOR expression. This is in contrast to previously reported synergistic effects of isoflavones and or other flavonoids on GAG synthesis inhibition^5^,^9^,^17^. We conclude that among two mechanisms of modulation of GAG metabolism by flavonoids, the inhibition of GAG synthesis is regulated by various pathways, depending on the kind of flavonoid, which may result in a cumulative effect if mixtures of two or more active compounds are used, as reported previously. In contrast, stimulation of lysosomal biogenesis (including enhancement of expression of genes coding for GAG-degrading enzymes) by different flavonoids (providing they are active in this process) may proceed according to the same mechanism, based on modulation of TFEB and MTOR levels. Therefore, combination of two flavonoids does not result in a synergistic effect in this regulatory pathway.

## Supplementary Material

Supplementary Information Table S1

## Figures and Tables

**Table 1:** Relative levels of GAG synthesis in HDFa and MPS II cells after 72 h treatment with various flavonoids (100 *µ*M genistein, 100 *µ*M kaempferol, 100 *µ*M daidzein, and mixtures of them of 30 *µ*M each) measured by incorporation of 3H-GlcN. Statistically significant differences in GAG production levels relative to control cells (treated with DMSO only) are indicated with * for p < 0.005, ** for p < 0.001, ns – not significant

Flavonoids	3H-GlcN incorporation relative to untreated control cells [%]			
	HDFa		MPS II	
	Mean value	SD	Mean value	SD
None (DMSO only)	100	7.6	100	9.5
Genistein	54**	8.6	44**	4.6
Kaempferol	35**	2.8	29**	3.2
Daidzein	115*	6.1	97^ns^	16.2
Genistein + Kaempferol	33**	6.9	46**	3.0
Genistein + Daidzein	65**	8.5	83*	8.8

**Table 2:** Number of genes revealing altered expression in response to various flavonoids' treatment type (genistein, kaempferol, daidzein, and mixtures of them as stated in the table), identified in the microarray analysis of whole genome sequences (0.5 ≥ FC ≥ 2.0), and GAG associated transcripts (0.7 ≥ FC ≥ 1.3) of HDFa cells, with the p-value < 0.05, and n ≥ 3

Gene expression modulation		No. of genes																			
		Genistein1)						Kaempferol						Genistein + Kaempferol		Daidzein				Genistein + Daidzein	
		Concentration [*μ*M]																			
		30		60		100		30		60		100		30 + 30		60		100		30 + 30	
		Time of exposure [h]																			
		24	48	24	48	24	48	24	48	24	48	24	48	24	48	24	48	24	48	24	48
**Whole genome**	**in sum**	33	59	260	370	468	661	551	504	340	975	698	1506	362	1328	102	519	57	555	84	601
	**up-regulation**	12	25	111	155	232	291	286	284	220	538	421	812	209	496	75	287	26	112	38	139
	**down-regulation**	21	34	149	215	236	370	265	220	120	437	277	694	153	832	27	232	31	443	46	462
**GAG synthesis**	**in sum**	1	0	10	5	9	7	5	4	3	7	8	13	6	8	1	5	1	11	3	8
	**up- regulation**	1	0	6	4	5	4	1	4	0	4	3	8	4	6	0	2	0	5	1	6
	**down- regulation**	0	0	4	1	4	3	4	0	3	3	5	5	2	2	1	3	1	6	2	2
**GAG degradation**	**in sum**	0	0	2	2	7	2	0	3	1	4	3	7	2	4	1	4	0	1	0	0
	**up- regulation**	0	0	2	2	7	2	0	3	1	4	3	6	2	4	1	3	0	0	0	0
	**down- regulation**	0	0	0	0	0	0	0	0	0	0	0	1	0	0	0	1	0	1	0	0

^1)^Results based on raw data from Moskot *et al.* (2014).

**Table 3:** GAG metabolism genes of HDFa after 24 and 48 h treatment with various flavonoids (100 *µ*M genistein, 100 *µ*M kaempferol, 100 *µ*M daidzein, and mixtures of them of 30 *µ*M each) identified in the microarray analysis (0.7 ≥ FC ≥ 1.3, n ≥ 3, with the p-value < 0.05). Cells shaded in red stand for up-regulation, while in blue for down-regulation

GAG metabolism		Genes	Compounds									
			Genistein1)		Kaempferol		Genistein + Kaempferol		Daidzein		Genistein + Daidzein	
			Time of exposure [h]									
			24	48	24	48	24	48	24	48	24	48
**GAG synthesis**	**Chain initiation**	*B3GAT3*										
		*B4GALT7*										
		*XYLT1*										
	**Chain polymerization**	*B3GNT2*										
		*B4GALT1*										
		*B4GALT4*										
		*CHPF*										
		*CHSY3*										
		*CSGALNACT2*										
		*EXT1*										
		*EXT2*										
		*EXTL2*										
		*EXTL3*										
		*ST3GAL1*										
		*ST3GAL2*										
		*ST3GAL3*										
	**Chain modification**	*CHST12*										
		*CHST14*										
		*CHST2*										
		*CHST3*										
		*CHST7*										
		*GLCE*										
		*HS2ST1*										
		*HS3ST3A1*										
		*NDST1*										
		*UST*										
**GAG degradation**		*GALNS*										
		*GNS*										
		*GUSB*										
		*HEXA*										
		*HEXB*										
		*HYAL3*										
		*IDUA*										
		*NAGLU*										
		*SGSH*										

1)Results based on raw data from Moskot *et al.* (2014).

**Table 4:** Expression patterns of GAG metabolism- and lysosome-associated genes in flavonoids (100 *µ*M genistein, 100 *µ*M kaempferol, and mixture of them of 30 *µ*M each, for 24 h) treated HDFa and MPS II fibroblasts analyzed with microarray and real-time qRT-PCR custom panel. Values represent fold change and denote differences for samples treated with tested compound or their mixture, against untreated samples, with respect to *TRFC* reference of constant expression level. Bolded texts are names of enzymes and genes belonging to GAG metabolism pathways

			Genistein			Kaempferol			Genistein + Kaempferol		
			FC ± SD			FC ± SD			FC ± SD		
			Microarray	Real-time qRT-PCR		Microarray	Real-time qRT-PCR		Microarray	Real-time qRT-PCR	
Category	Enzyme	Gene	HDFa		MPS II	HDFa		MPS II	HDFa		MPS II
GAG synthesis proteins	**chondroitin polymerizing factor**	***CHPF***	1.3 ± 0.5	1.3 ± 0.2	1.5 ± 0.0	1.6 ± 0.4	1.1 ± 0.1	1.7 ± 0.1	1.5 ± 0.2	1.1 ± 0.3	0.9 ± 0.2
	**chondroitin sulfate synthase 3**	***CHSY3***	0.9 ± 0.2	0.7 ± 0.1	0.5 ± 0.0	1.1 ± 0.1	0.7 ± 0.1	1.0 ± 0.1	1.4 ± 0.2	0.8 ± 0.0	0.8 ± 0.1
	**exostosin glycosyltransferase 1**	***EXT1***	0.6 ± 0.2	1.0 ± 0.1	0.6 ± 0.1	0.7 ± 0.1	0.9 ± 0.1	0.8 ± 0.0	0.7 ± 0.0	0.7 ± 0.2	0.7 ± 0.1
	**heparan sulfate sulfotransferase 3A1**	***HS3ST3A1***	0.5 ± 0.2	0.7 ± 0.1	0.3 ± 0.0	0.6 ± 0.2	1.3 ± 0.2	0.6 ± 0.0	0.8 ± 0.1	1.2 ± 0.2	1.0 ± 0.2
	**sialyltransferase 4B**	***ST3GAL2***	0.6 ± 0.1	0.4 ± 0.0	0.4 ± 0.0	1.0 ± 0.1	0.6 ± 0.1	0.8 ± 0.0	0.8 ± 0.1	0.5 ± 0.2	0.6 ± 0.1
	**sialyltransferase 6**	***ST3GAL3***	1.1 ± 0.1	1.4 ± 0.2	1.0 ± 0.1	0.7 ± 0.1	1.0 ± 0.1	0.9 ± 0.1	0.9 ± 0.1	0.8 ± 0.2	0.8 ± 0.2
	**xylosyltransferase I**	***XYLT1***	0.6 ± 0.2	0.3 ± 0.0	0.3 ± 0.0	0.8 ± 0.1	0.4 ± 0.0	0.6 ± 0.1	n.d.	0.4 ± 0.1	0.4 ± 0.1
Lysosomal proteins	ATP-binding cassette, sub-family A (ABC1), member 9	*ABCA9*	1.1 ± 0.4	2.8 ± 0.6	1.8 ± 0.4	1.2 ± 0.0	1.3 ± 0.4	0.7 ± 0.1	1.4 ± 0.1	0.8 ± 0.2	0.8 ± 0.2
	acid phosphatase 2	*ACP2*	1.5 ± 0.2	2.1 ± 0.4	1.0 ± 0.0	1.2 ± 0.0	1.1 ± 0.1	0.9 ± 0.1	1.4 ± 0.5	0.9 ± 0.2	0.8 ± 0.1
	acid phosphatase 5	*ACP5*	1.6 ± 0.1	3.1 ± 0.4	1.3 ± 0.0	1.9 ± 0.0	1.9 ± 0.2	1.2 ± 0.3	1.6 ± 0.1	1.4 ± 0.2	1.6 ± 0.8
	aspartylglucosaminidase	*AGA*	1.9 ± 0.5	3.2 ± 0.4	1.6 ± 0.2	1.5 ± 0.0	2.1 ± 0.3	1.3 ± 0.1	1.2 ± 0.1	1.6 ± 0.3	1.4 ± 0.3
	adaptor-related protein complex 3, sigma 2 subunit	*AP3S2*	1.4 ± 0.3	1.3 ± 0.2	1.0 ± 0.3	1.6 ± 0.1	1.1 ± 0.1	1.0 ± 0.1	1.4 ± 0.1	1.0 ± 0.1	0.9 ± 0.2
	arylsulfatase A	*ARSA*	1.3 ± 0.2	2.3 ± 0.3	1.7 ± 0.2	1.0 ± 0.0	1.3 ± 0.1	1.4 ± 0.1	1.4 ± 0.2	1.1 ± 0.0	1.0 ± 0.1
	arylsulfatase G	*ARSG*	1.2 ± 0.4	5.1 ± 1.1	2.1 ± 0.2	3.4 ± 0.0	6.6 ± 0.8	1.3 ± 0.2	1.2 ± 0.1	1.7 ± 0.2	1.5 ± 0.4
	acid ceramidase 1	*ASAH1*	1.7 ± 0.8	2.5 ± 0.4	1.5 ± 0.1	1.2 ± 0.0	1.5 ± 0.2	1.5 ± 0.1	1.1 ± 0.0	1.0 ± 0.1	1.0 ± 0.1
	ceroid-lipofuscinosis, neuronal protein 3	*CLN3*	1.4 ± 0.4	1.3 ± 0.2	1.1 ± 0.0	1.3 ± 0.0	1.5 ± 0.2	1.6 ± 0.0	2.0 ± 0.1	0.9 ± 0.2	0.8 ± 0.1
	ceroid-lipofuscinosis neuronal protein 5	*CLN5*	2.5 ± 0.3	3.4 ± 0.4	1.5 ± 0.2	2.4 ± 0.0	2.2 ± 0.2	1.5 ± 0.0	1.5 ± 0.1	1.4 ± 0.3	1.3 ± 0.2
	cystinosin	*CTNS*	1.5 ± 0.4	1.1 ± 0.2	0.9 ± 0.0	1.7 ± 0.0	1.5 ± 0.1	1.5 ± 0.1	1.1 ± 0.1	0.8 ± 0.1	0.8 ± 0.1
	cathepsin A	*CTSA*	1.5 ± 0.3	2.4 ± 0.3	1.2 ± 0.1	1.1 ± 0.0	1.5 ± 0.1	1.2 ± 0.1	1.0 ± 0.0	1.0 ± 0.1	0.9 ± 0.2
	cathepsin D	*CTSD*	1.2 ± 0.4	1.6 ± 0.2	1.4 ± 0.2	1.1 ± 0.0	1.4 ± 0.2	1.5 ± 0.1	n.d.	1.0 ± 0.1	1.1 ± 0.3
	cathepsin F	*CTSF*	1.7 ± 0.5	2.6 ± 0.4	1.6 ± 0.3	1.7 ± 0.0	2.0 ± 0.2	1.8 ± 0.2	n.d.	1.2 ± 0.2	1.4 ± 0.3
	cathepsin K	*CTSK*	1.2 ± 0.2	3.2 ± 0.5	1.7 ± 0.2	1.1 ± 0.0	2.2 ± 0.3	1.6 ± 0.1	0.8 ± 0.1	1.2 ± 0.3	0.9 ± 0.2
	cathepsin O	*CTSO*	1.6 ± 0.4	3.1 ± 0.3	1.5 ± 0.2	1.1 ± 0.0	1.8 ± 0.1	1.1 ± 0.1	n.d.	1.1 ± 0.2	1.0 ± 0.3
	deoxyribonuclease-2-alpha	*DNASE2*	1.1 ± 0.1	2.1 ± 0.4	1.0 ± 0.1	2.3 ± 0.0	2.5 ± 0.3	1.5 ± 0.1	n.d.	1.1 ± 0.3	1.0 ± 0.3
	alpha glucosidase	*GAA*	1.6 ± 0.7	1.4 ± 0.1	1.8 ± 0.2	1.2 ± 0.0	1.0 ± 0.1	1.4 ± 0.1	1.1 ± 0.1	0.9 ± 0.3	0.9 ± 0.2
	**N-acetylgalactosamine-6-sulfatase**	***GALNS***	1.1 ± 0.2	1.4 ± 0.2	1.1 ± 0.1	1.2 ± 0.0	1.0 ± 0.2	1.2 ± 0.1	1.3 ± 0.1	1.0 ± 0.2	0.9 ± 0.2
	**N-acetylglucosamine-6-sulfatase**	***GNS***	1.2 ± 0.2	1.4 ± 0.1	1.1 ± 0.0	1.2 ± 0.1	1.0 ± 0.1	1.2 ± 0.0	1.2 ± 0.1	0.8 ± 0.0	0.8 ± 0.0
	**hexosaminidase A**	***HEXA***	1.4 ± 0.2	2.2 ± 0.3	1.7 ± 0.2	1.4 ± 0.3	1.4 ± 0.2	1.4 ± 0.1	1.3 ± 0.3	1.0 ± 0.1	0.9 ± 0.1
	**hyaluronoglucosaminidase 3**	***HYAL3***	1.7 ± 0.5	1.2 ± 0.3	0.7 ± 0.2	1.4 ± 0.2	1.2 ± 0.2	1.4 ± 0.0	0.9 ± 0.0	1.0 ± 0.1	0.9 ± 0.2
	**alpha L-iduronidase**	***IDUA***	1.4 ± 0.3	1.0 ± 0.2	1.2 ± 0.1	1.3 ± 0.1	1.0 ± 0.2	1.5 ± 0.1	1.2 ± 0.0	0.9 ± 0.4	0.8 ± 0.3
	lysosomal-associated membrane protein 2	*LAMP2*	1.2 ± 0.1	1.7 ± 0.4	1.5 ± 0.2	1.1 ± 0.0	1.2 ± 0.2	0.9 ± 0.1	n.d.	1.0 ± 0.1	0.9 ± 0.1
	legumain	*LGMN*	1.4 ± 0.3	3.2 ± 0.5	1.4 ± 0.3	2.0 ± 0.0	2.1 ± 0.3	1.4 ± 0.1	1.4 ± 0.1	1.7 ± 0.4	1.4 ± 0.4
	lysosomal acid lipase	*LIPA*	1.1 ± 0.3	1.9 ± 0.3	1.1 ± 0.1	1.4 ± 0.0	1.0 ± 0.1	0.7 ± 0.1	1.1 ± 0.2	0.9 ± 0.1	0.9 ± 0.2
	alpha mannosidase	*MAN2B1*	1.3 ± 0.4	2.4 ± 0.1	1.3 ± 0.2	1.2 ± 0.0	1.8 ± 0.1	1.5 ± 0.1	1.7 ± 0.1	1.2 ± 0.2	1.1 ± 0.3
	beta mannosidase	*MANBA*	2.4 ± 0.8	3.8 ± 0.7	2.5 ± 0.1	1.5 ± 0.0	1.6 ± 0.2	1.6 ± 0.0	1.1 ± 0.1	1.3 ± 0.2	1.3 ± 0.3
	mucolipin 1	*MCOLN1*	1.2 ± 0.4	1.0 ± 0.1	1.0 ± 0.0	1.1 ± 0.0	0.8 ± 0.1	1.3 ± 0.1	n.d.	0.6 ± 0.0	0.6 ± 0.1
	**alpha-N-acetylglucosaminidase**	***NAGLU***	1.6 ± 0.5	1.2 ± 0.4	1.6 ± 0.3	1.2 ± 0.3	1.3 ± 0.5	0.9 ± 0.2	1.3 ± 0.1	1.0 ± 0.2	0.8 ± 0.3
	sialidase 1	*NEU1*	2.9 ± 0.4	4.1 ± 0.6	2.6 ± 0.2	1.6 ± 0.0	1.9 ± 0.2	1.7 ± 0.1	1.4 ± 0.2	1.3 ± 0.2	1.1 ± 0.2
	Niemann-Pick C1-like protein 1	*NPC1*	1.4 ± 0.4	1.5 ± 0.2	0.9 ± 0.0	1.4 ± 0.0	2.3 ± 0.1	1.0 ± 0.1	1.2 ± 0.1	1.5 ± 0.1	1.4 ± 0.3
	mitochondrial carrier 1	*PSAP*	1.3 ± 0.0	2.3 ± 0.2	1.5 ± 0.2	1.2 ± 0.0	1.7 ± 0.1	1.5 ± 0.1	n.d.	1.0 ± 0.2	1.0 ± 0.2
	lysosome membrane protein 2	*SCARB2*	1.2 ± 0.2	1.9 ± 0.2	0.9 ± 0.1	1.2 ± 0.0	1.3 ± 0.1	1.1 ± 0.1	1.0 ± 0.1	1.1 ± 0.2	1.0 ± 0.2
	sphingomyelin phosphodiesterase 1	*SMPD1*	1.4 ± 0.2	1.9 ± 0.3	1.3 ± 0.0	1.0 ± 0.0	1.2 ± 0.1	1.2 ± 0.1	0.9 ± 0.1	1.0 ± 0.1	0.9 ± 0.1
	sulfatase modifying factor 1	*SUMF1*	1.6 ± 0.2	2.5 ± 0.4	1.7 ± 0.3	1.4 ± 0.0	1.5 ± 0.1	1.4 ± 0.1	1.3 ± 0.1	1.0 ± 0.3	0.9 ± 0.2

n.d. stands for not detected.

**Table 5:** GSEA analysis of selective gene sets enriched among genes up-and down-regulated by tested flavonoids (100 *µ*M genistein, 100 *µ*M kaempferol, 100 *µ*M daidzein, and mixtures of them of 30 *µ*M each, for 24 and 48 hours) in HDFa cells

Enrichment		Genistein		Kaempferol		Genistein + Kaempferol		Daidzein		Genistein + Daidzein	
		Time of exposure [h]									
		24	48	24	48	24	48	24	48	24	48
**Up-regulation**	Gene sets significant in phenotype	73	73	86	71	80	109	115	86	95	99
	Gene sets significant at FDR < 25%	38	22	33	9	35	35	7	14	13	24
	Gene sets significantly enriched at nominal p-value < 1%	13	8	15	5	15	16	4	5	6	14
**Down-regulation**	Gene sets significant in phenotype	105	105	92	107	98	69	63	92	83	79
	Gene sets significant at FDR < 25%	54	48	27	56	24	15	0	13	1	4
	Gene sets significantly enriched at nominal p-value < 1%	31	31	13	29	14	14	0	9	4	5
