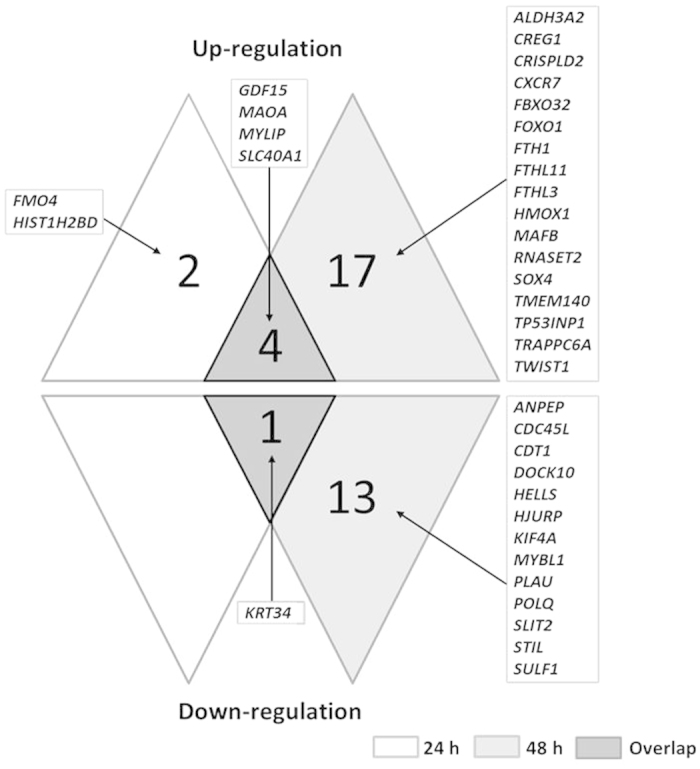# Corrigendum: Modulation of expression of genes involved in glycosaminoglycan metabolism and lysosome biogenesis by flavonoids

**DOI:** 10.1038/srep22809

**Published:** 2016-03-31

**Authors:** Marta Moskot, Joanna Jakóbkiewicz-Banecka, Anna Kloska, Elwira Smolińska, Paweł Mozolewski, Marcelina Malinowska, Michał Rychłowski, Bogdan Banecki, Grzegorz Węgrzyn, Magdalena Gabig-Cimińska

Scientific Reports 5: Article number: 937810.1038/srep09378; published online: 03232015; updated: 03312016

This Article contains an error in Figure 3, where the numbers of genes are incorrectly indicated in the 24-hour and 48-hour sections. The correct Figure 3 appears below as Fig. 1.

## Figures and Tables

**Figure 1:**