# Corrigendum: Modulation of expression of genes involved in glycosaminoglycan metabolism and lysosome biogenesis by flavonoids

**DOI:** 10.1038/srep38118

**Published:** 2016-12-09

**Authors:** Marta Moskot, Joanna Jakóbkiewicz-Banecka, Anna Kloska, Elwira Smolińska, Paweł Mozolewski, Marcelina Malinowska, Michał Rychłowski, Bogdan Banecki, Grzegorz Węgrzyn, Magdalena Gabig-Cimińska

Scientific Reports 5: Article number: 937810.1038/srep09378; published online: 03 23 2015; updated: 12 09 2016

This acknowledgments section in this Article is incomplete:

“This work was supported by National Science Centre (project grant no. UMO-2011/01/B/NZ1/03686 and N N301 668540)”.

should read:

“This work was supported by National Science Centre (project grant no. UMO-2011/01/B/NZ1/03686 and N N301 668540). Marta Moskot was funded by National Science Centre project grant no. UMO-2014/12/T/NZ2/00538”.

