# Supplementary material for: Modulation of expression of genes involved in glycosaminoglycan metabolism and lysosome biogenesis by flavonoids
Source: Sci Rep. 2015 Mar 23;5:9378. doi: 10.1038/srep09378 (PMC4369724; doi:10.1038/srep09378)
Supplement: Supplementary Information — Table S1 [file srep09378-s1.docx]

**Modulation of expression of genes involved in glycosaminoglycan metabolism and lysosome biogenesis by flavonoids**

Marta Moskot^a^, Joanna Jakóbkiewicz-Banecka^b^, Anna Kloska^b^, Elwira Smolińska^b^, Paweł Mozolewski^b^, Marcelina Malinowska^b^, Michał Rychłowski^c^, Bogdan Banecki^d^, Grzegorz Węgrzyn^b^, Magdalena Gabig-Cimińska^a,*^

^a^*Laboratory of Molecular Biology (affiliated with the University of Gdańsk), Institute of Biochemistry and Biophysics, Polish Academy of Sciences, Wita Stwosza 59, 80-308 Gdańsk, Poland*

^b^*Department of Molecular Biology, University of Gdańsk, Wita Stwosza 59, 80-308 Gdańsk, Poland*

^c^*Department of Molecular Virology*, *Intercollegiate Faculty of Biotechnology UG-MUG,* *Kładki 24, 80-822 Gdańsk, Poland*

^d^*Department of Molecular and Cellular Biology*, *Intercollegiate Faculty of Biotechnology UG-MUG,* *Kładki 24, 80-822 Gdańsk, Poland*

^*^Corresponding author:

Laboratory of Molecular Biology (affiliated with the University of Gdańsk), Institute of Biochemistry and Biophysics, Polish Academy of Sciences, Wita Stwosza 59, 80-308 Gdańsk, Poland, Tel. +48 58 523 6046, fax: +48 58 523 6025; E-mail: [m.gabig@biol.ug.edu.pl](mailto:m.gabig@biol.ug.edu.pl)

Table S1. Relative levels of GAG in HDFa and MPS II cells after 10-days treatment with various flavonoids (100 *µ*M genistein, 100 *µ*M kaempferol, 100 *µ*M daidzein, and mixtures of them of 30 *µ*M each).

| **Flavonoids** | **GAG amount relative to untreated control cells [%]** | | | |
| --- | --- | --- | --- | --- |
|  | **HDFa** | | **MPS II** | |
|  | **Mean value** | **SD** | **Mean value** | **SD** |
| None (DMSO only) | 100 | 3.6 | 100 | 6.7 |
| Genistein | 40 | 8.6 | 81 | 20.6 |
| Kaempferol | 26 | 1.6 | 63 | 26.7 |
| Daidzein | 51 | 10.8 | 58 | 8.1 |
| Genistein + Kaempferol | 70 | 14.0 | 53 | 12.6 |
| Genistein + Daidzein | 59 | 7.8 | 85 | 15.1 |
